# Supplementary material for: Multiomics characterisation of the zoo-housed gorilla gut microbiome reveals bacterial community compositions shifts, fungal cellulose-degrading, and archaeal methanogenic activity
Source: Gut Microbiome (Camb). 2023 Jul 19;4:e12. doi: 10.1017/gmb.2023.11 (PMC11406404; doi:10.1017/gmb.2023.11)
Supplement: Supplementary file 1 [file S2632289723000117sup001.zip › S2632289723000117sup001.docx]

**Supplementary Figure S1**

**Manuscript:**

Houtkamp, I., Van Zijll Langhout, M., Bessem, M., Pirovano, W., & Kort, R. (2023). Multiomics characterization of the of the zoo-housed gorilla gut microbiome reveals bacterial community compositions shifts, fungal cellulose-degrading, and archaeal methanogenic activity. *Gut Microbiome,* 1-25. doi:10.1017/gmb.2023.11


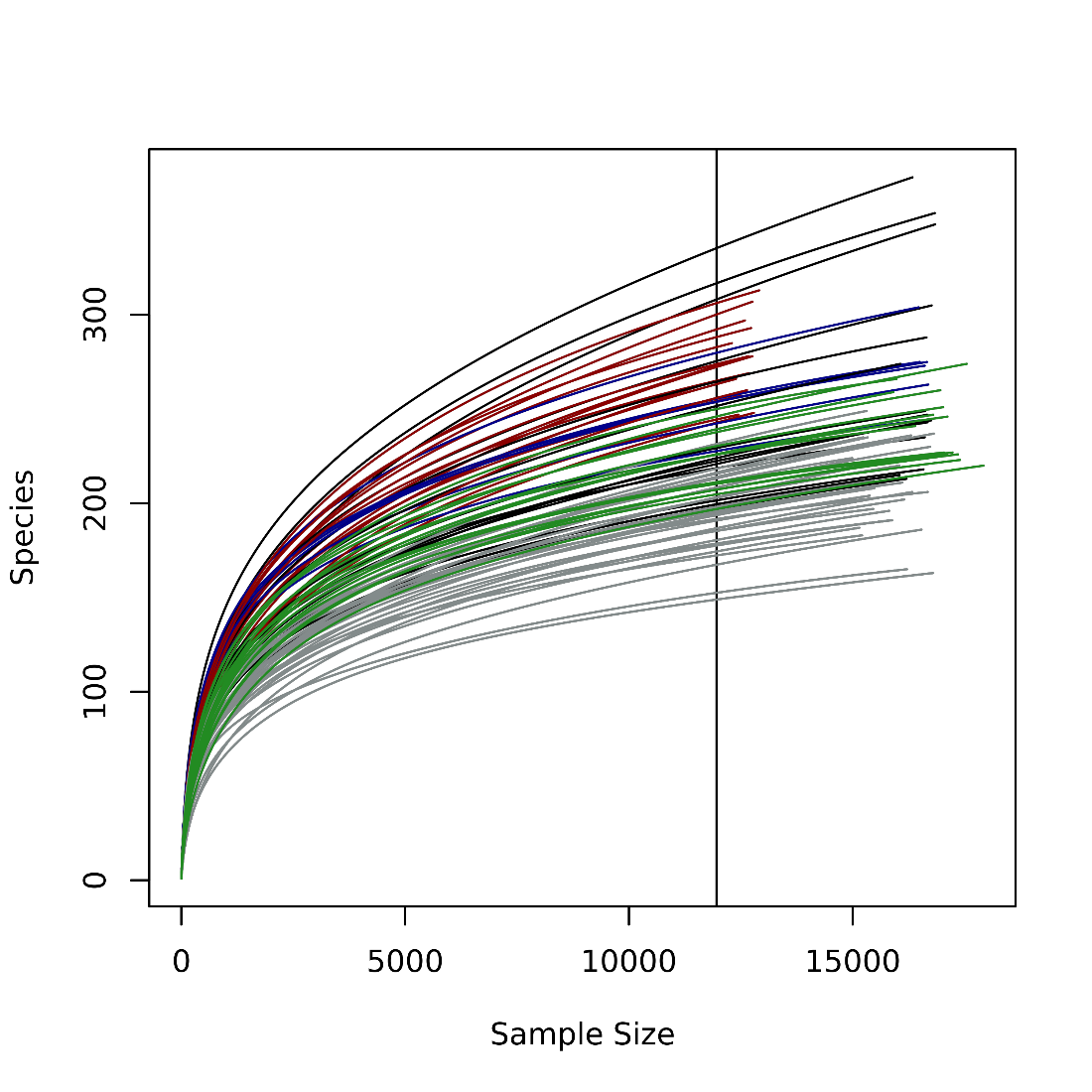


**Figure S1: Rarefaction curve of 16S rRNA gene amplicon sequence data.** Microbiota of zoo-housed gorillas, red (this study); Microbiota of wild gorillas, blue (Campbell et al.,2020); Microbiota of zoo-housed gorillas, green (Campbell *et al.*,2020); Microbiota of wild gorillas, black (Narat *et al.*,2020); Microbiota of zoo-housed gorillas, grey (Narat et al.,2020).
