# Supplementary material for: Multiomics characterisation of the zoo-housed gorilla gut microbiome reveals bacterial community compositions shifts, fungal cellulose-degrading, and archaeal methanogenic activity
Source: Gut Microbiome (Camb). 2023 Jul 19;4:e12. doi: 10.1017/gmb.2023.11 (PMC11406404; doi:10.1017/gmb.2023.11)
Supplement: Supplementary file 1 [file S2632289723000117sup001.zip › S2632289723000117sup002.docx]

**Supplementary Figure S2**

**Manuscript:**

Houtkamp, I., Van Zijll Langhout, M., Bessem, M., Pirovano, W., & Kort, R. (2023). Multiomics characterization of the of the zoo-housed gorilla gut microbiome reveals bacterial community compositions shifts, fungal cellulose-degrading, and archaeal methanogenic activity. *Gut Microbiome,* 1-25. doi:10.1017/gmb.2023.11


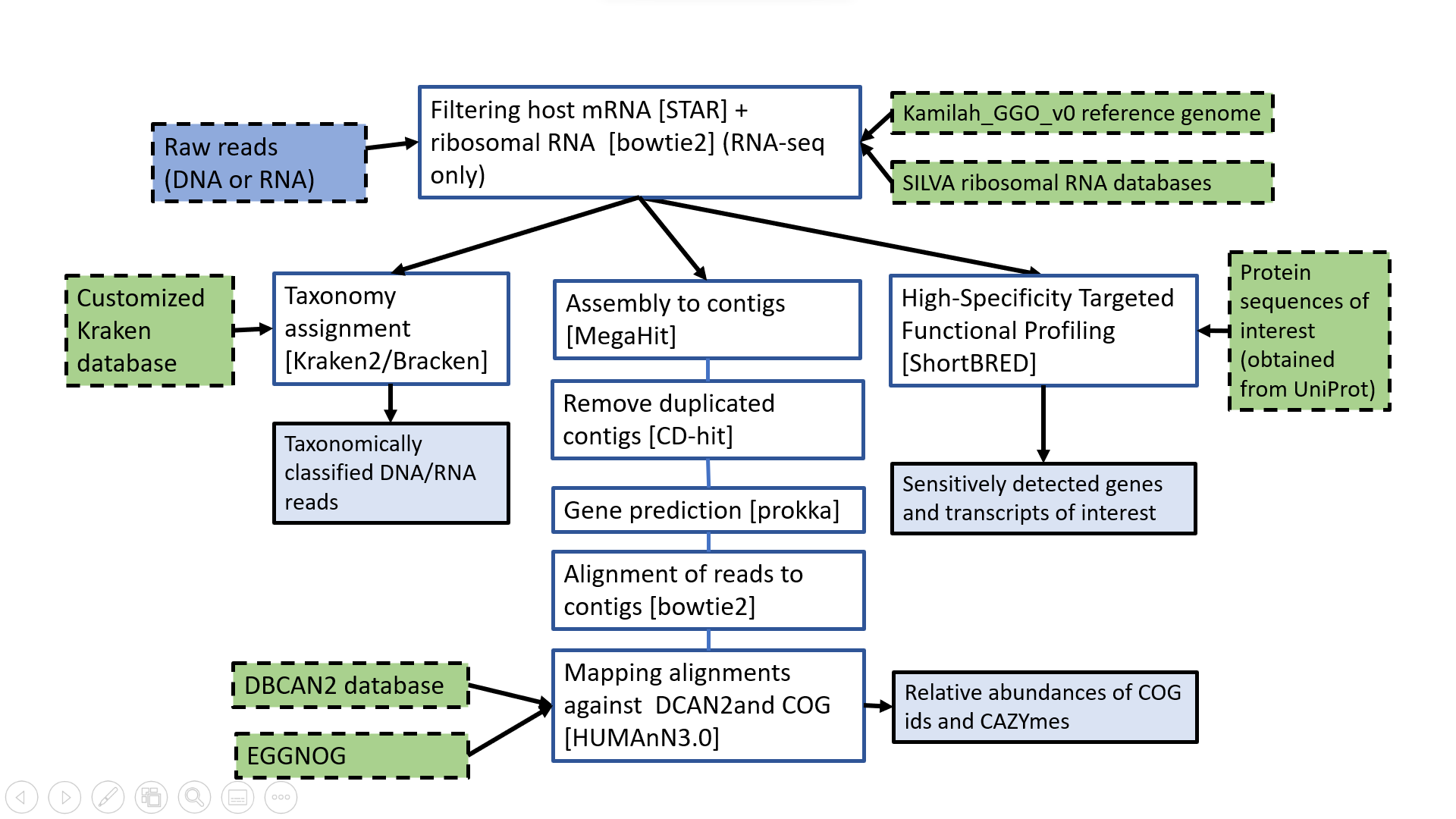


**Figure S2: Shotgun metagenomics/RNA-seq analysis workflow.** The workflow summarizes the analysis of DNA and RNA reads obtained through shotgun metagenomics and RNA-seq, respectively, as described in detail in the methods section.
