# Supplementary material for: Multiomics characterisation of the zoo-housed gorilla gut microbiome reveals bacterial community compositions shifts, fungal cellulose-degrading, and archaeal methanogenic activity
Source: Gut Microbiome (Camb). 2023 Jul 19;4:e12. doi: 10.1017/gmb.2023.11 (PMC11406404; doi:10.1017/gmb.2023.11)
Supplement: Supplementary file 1 [file S2632289723000117sup001.zip › S2632289723000117sup005.docx]

**Supplementary Figure S5**

**Manuscript:**

Houtkamp, I., Van Zijll Langhout, M., Bessem, M., Pirovano, W., & Kort, R. (2023). Multiomics characterization of the of the zoo-housed gorilla gut microbiome reveals bacterial community compositions shifts, fungal cellulose-degrading, and archaeal methanogenic activity. *Gut Microbiome,* 1-25. doi:10.1017/gmb.2023.11


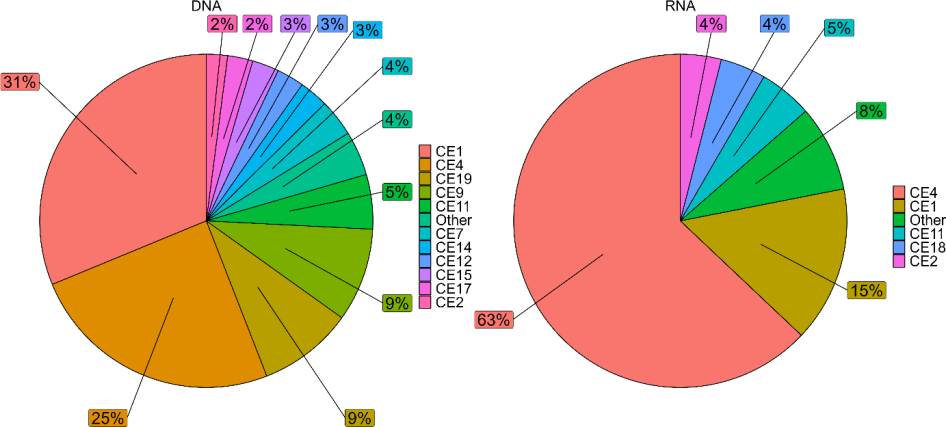


**Figure S5: CE families in the gut metagenome and metatranscriptome of a zoo-housed gorilla (DNA vs RNA).** Percentages represent the percentual contribution of each Carbohydrate esterase (CE) family to the total number of CE families found by shotgun metagenomics (DNA, left) and RNA-seq (RNA, right) on fecal samples of a zoo-housed gorilla.
