# Supplementary material for: Multiomics characterisation of the zoo-housed gorilla gut microbiome reveals bacterial community compositions shifts, fungal cellulose-degrading, and archaeal methanogenic activity
Source: Gut Microbiome (Camb). 2023 Jul 19;4:e12. doi: 10.1017/gmb.2023.11 (PMC11406404; doi:10.1017/gmb.2023.11)
Supplement: Supplementary file 1 [file S2632289723000117sup001.zip › S2632289723000117sup007.docx]

**Supplementary Table S1**

**Manuscript:**

Houtkamp, I., Van Zijll Langhout, M., Bessem, M., Pirovano, W., & Kort, R. (2023). Multiomics characterization of the of the zoo-housed gorilla gut microbiome reveals bacterial community compositions shifts, fungal cellulose-degrading, and archaeal methanogenic activity. *Gut Microbiome,* 1-25. doi:10.1017/gmb.2023.11

| Phylum | Zoo-housed gorillas | Wild gorillas |
| --- | --- | --- |
| Firmicutes | 47.2% | 45.4% |
| Bacteroidetes | 30.2% | 14.9% |
| Spirochaetes | 13.4% | 2.4% |
| Actinobacteria | 4.0% | 14.2% |
| Proteobacteria | 2.7% | 6.5% |
| Chloroflexi | 0.13% | 15.1% |

**Table S1: Relative abundances of the top 6 bacterial phyla in zoo-housed gorilla and wild gorilla samples based on 16s rRNA amplicon sequencing.**
