# Supplementary material for: Multiomics characterisation of the zoo-housed gorilla gut microbiome reveals bacterial community compositions shifts, fungal cellulose-degrading, and archaeal methanogenic activity
Source: Gut Microbiome (Camb). 2023 Jul 19;4:e12. doi: 10.1017/gmb.2023.11 (PMC11406404; doi:10.1017/gmb.2023.11)
Supplement: Supplementary file 1 [file S2632289723000117sup001.zip › S2632289723000117sup008.docx]

**Supplementary Table S2**

**Manuscript:**

Houtkamp, I., Van Zijll Langhout, M., Bessem, M., Pirovano, W., & Kort, R. (2023). Multiomics characterization of the of the zoo-housed gorilla gut microbiome reveals bacterial community compositions shifts, fungal cellulose-degrading, and archaeal methanogenic activity. *Gut Microbiome,* 1-25. doi:10.1017/gmb.2023.11

|  | **Top 10 genera in zoo- housed gorilla samples** | **Relative abundance (%)** | **Top 10 genera in wild gorilla samples** | **Relative** abundance **(%)** |
| --- | --- | --- | --- | --- |
| 1 | *Treponema* | 12.7 | *Flexilinea* | 15.0 |
| 2 | *Prevotella* | 8.7 | *Eubacterium* | 5.7 |
| 3 | *Lentimicrobium* | 7.9 | *Prevotella* | 5.6 |
| 4 | *Lactobacillus* | 6.8 | *Acinetobacter* | 4.2 |
| 5 | *Sporobacter* | 3.5 | *Parvibacter* | 4.1 |
| 6 | *Clostridium* | 3.2 | *unclassified Bacteroidales* | 3.2 |
| 7 | *Intestinimonas* | 2.6 | *Senegalimassilia* | 3.1 |
| 8 | *Ruminococcus* | 2.4 | *unclassified Eubacteriaceae* | 2.7 |
| 9 | *Parabacteroides* | 2.2 | *Clostridium* | 2.6 |
| 10 | *Oscillospira* | 2.1 | *Mogibacterium* | 2.6 |

**Table S2: Relative abundances of the top 10 bacterial genera in zoo-housed gorilla and wild gorilla samples.**
