# Supplementary material for: Multiomics characterisation of the zoo-housed gorilla gut microbiome reveals bacterial community compositions shifts, fungal cellulose-degrading, and archaeal methanogenic activity
Source: Gut Microbiome (Camb). 2023 Jul 19;4:e12. doi: 10.1017/gmb.2023.11 (PMC11406404; doi:10.1017/gmb.2023.11)
Supplement: Supplementary file 1 [file S2632289723000117sup001.zip › S2632289723000117sup011.docx]

**Supplementary Table S5**

**Manuscript:**

Houtkamp, I., Van Zijll Langhout, M., Bessem, M., Pirovano, W., & Kort, R. (2023). Multiomics characterization of the of the zoo-housed gorilla gut microbiome reveals bacterial community compositions shifts, fungal cellulose-degrading, and archaeal methanogenic activity. *Gut Microbiome,* 1-25. doi:10.1017/gmb.2023.11

|  | **Top 10 archaeal genera in zoo- housed metatranscriptome** | **Relative abundance (%)** |
| --- | --- | --- |
| 1 | *Methanobrevibacter* | 8.3 |
| 2 | *Candidatus Methanoplasma* | 6.8 |
| 3 | *Candidatus Methanomethylophilus* | 3.8 |
| 4 | *Methanomassiliicoccus* | 0.4 |
| 5 | *Methanobacterium* | <0.1 |
| 6 | *Methanoregula* | <0.1 |
| 7 | *Halorussus* | <0.1 |
| 8 | *Candidatus Methanoperedens* | <0.1 |
| 9 | *Cuniculiplasma* | <0.1 |
| 10 | *Candidatus Micrarchaeum* | <0.1 |

**Table S5: Relative abundances of the top 10 archaeal eukaryotic genera in zoo-housed gorilla gut metatranscriptome.**
