# Supplementary material for: Multiomics characterisation of the zoo-housed gorilla gut microbiome reveals bacterial community compositions shifts, fungal cellulose-degrading, and archaeal methanogenic activity
Source: Gut Microbiome (Camb). 2023 Jul 19;4:e12. doi: 10.1017/gmb.2023.11 (PMC11406404; doi:10.1017/gmb.2023.11)
Supplement: Supplementary file 1 [file S2632289723000117sup001.zip › S2632289723000117sup012.docx]

**Supplementary Table S6**

**Manuscript:**

Houtkamp, I., Van Zijll Langhout, M., Bessem, M., Pirovano, W., & Kort, R. (2023). Multiomics characterization of the of the zoo-housed gorilla gut microbiome reveals bacterial community compositions shifts, fungal cellulose-degrading, and archaeal methanogenic activity. *Gut Microbiome,* 1-25. doi:10.1017/gmb.2023.11

| **Class** | **Function** |
| --- | --- |
| Glycoside hydrolases (GH) | Hydrolysis and/or transglycosylation of glycosidic bonds. |
| Glycosyltransferases (GT) | Biosynthesis of glycosidic bonds from phospho-activated sugar donors. |
| Polysaccharide lyases (PL) | Cleavage of the glycosidic bonds of uronic acid-containing polysaccharides by a β-elimination mechanism. |
| Carbohydrate esterases (CE) | Removal of ester-based modifications present in mono-, oligo- and polysaccharides, thereby facilitating the action of GHs on complex polysaccharides. |
| Carbohydrate-binding modules (CBM) | Targeted to substrate and promoting prolonged interacting, thereby potentiating the enzymatic activities of CAZymes. |
| Auxiliary Activities (AA) | Catalytic enzymes potentially involved in plant cell degradation through an ability to help the original GH, PL and CE enzymes gain access to the carbohydrates comprising the plant cell wall |

**Table S6: CAZyDB classes and their associated function.**
