# Supplementary material for: Multiomics characterisation of the zoo-housed gorilla gut microbiome reveals bacterial community compositions shifts, fungal cellulose-degrading, and archaeal methanogenic activity
Source: Gut Microbiome (Camb). 2023 Jul 19;4:e12. doi: 10.1017/gmb.2023.11 (PMC11406404; doi:10.1017/gmb.2023.11)
Supplement: Supplementary file 1 [file S2632289723000117sup001.zip › S2632289723000117sup013.docx]

**Supplementary Table S7**

**Manuscript:**

Houtkamp, I., Van Zijll Langhout, M., Bessem, M., Pirovano, W., & Kort, R. (2023). Multiomics characterization of the of the zoo-housed gorilla gut microbiome reveals bacterial community compositions shifts, fungal cellulose-degrading, and archaeal methanogenic activity. *Gut Microbiome,* 1-25. doi:10.1017/gmb.2023.11

| **UniProt ID** | **Samples** | **Name** | **Organism** | **UniProt Annotation status** |
| --- | --- | --- | --- | --- |
| **H2BPU6** | ALL | Putative cellulase | *Neocallimastix patriciarum* (rumen fungus) | Evidence at transcript level |
| **Q8J1E3** | ALL | Cellulase Cel48A | *Piromyces sp. (strain E2)* | Evidence at transcript level |
| **A0A220DC16** | ALL | Glycoside hydrolase family 48 protein | *uncultured actinobacterium (soil)* | Predicted |
| **R9T9W1** | 15_07_2_RNA,  09_08_1_RNA,  09_08_2_RNA | Glycoside hydrolase 48 family protein | *uncultured bacterium (rumen)* | Predicted |
| **R9T9K1** | 15_07_1_RNA, 15_07_2_RNA | Glycoside hydrolase 48 family protein | *uncultured bacterium (rumen)* | Predicted |
| **R9TAI7** | 15_07_2_RNA | Glycoside hydrolase 48 family protein | *uncultured bacterium (rumen)* | Predicted |
| **R9TCD2** | ALL | Glycoside hydrolase 48 family protein | *uncultured bacterium (rumen)* | Predicted |
| **R9TDC9** | 09_08_1_RNA, 09_08_2_RNA | Glycoside hydrolase 48 family protein | *uncultured bacterium (rumen)* | Predicted |
| **B0FEW0** | 15_07_1 RNA, 15_07_2 RNA | Cellobiohydrolase | *Piromyces rhizinflatus* | Evidence at transcript level |

**Table S7: GH6 and GH48 proteins detected in zoo-housed metatranscriptome.** Protein sequences belonging to families GH6 and GH48 detected with ShortBRED amongst RNA reads originating from the ZHG microbiome.
