# Supplementary material for: Multiomics characterisation of the zoo-housed gorilla gut microbiome reveals bacterial community compositions shifts, fungal cellulose-degrading, and archaeal methanogenic activity
Source: Gut Microbiome (Camb). 2023 Jul 19;4:e12. doi: 10.1017/gmb.2023.11 (PMC11406404; doi:10.1017/gmb.2023.11)
Supplement: Supplementary file 1 [file S2632289723000117sup001.zip › S2632289723000117sup014.docx]

**Supplementary Table S8**

**Manuscript:**

Houtkamp, I., Van Zijll Langhout, M., Bessem, M., Pirovano, W., & Kort, R. (2023). Multiomics characterization of the of the zoo-housed gorilla gut microbiome reveals bacterial community compositions shifts, fungal cellulose-degrading, and archaeal methanogenic activity. *Gut Microbiome,* 1-25. doi:10.1017/gmb.2023.11

| **UniProt ID** | **Samples** | **Name** | **Organism** | **UniProt Annotation status** |
| --- | --- | --- | --- | --- |
| **A0A1Y1WF22** | 15_07_1_RNA, 15_07_2_RNA | Homoaconitase, mitochondrial | *Anaeromyces robustus* | Unreviewed- protein inferred from homology |
| **A0A1Y1V9J2** | 15_07_2_RNA | Arg5,6 arginine biosynthetic enzyme | *Piromyces finnis* | Unreviewed- protein inferred from homology |
| **A0A1Y1WWZ0** | 15_07_1_RNA, 15_07_2_RNA,  09_08_1_RNA | Adenylate kinase | *Anaeromyces robustus* | Unreviewed- protein inferred from homology |
| **A0A1Y1XN91** | 15_07_2_RNA | Dihydroorotate dehydrogenase (quinone), mitochondrial | *Anaeromyces robustus* | Unreviewed- protein inferred from homology |
| **A0A1Y2CVY2** | 15_07_2_RNA | Aconitate hydratase, mitochondrial | *Neocallimastix californiae* | Unreviewed- protein inferred from homology |
| **A0A1Y2DRS2** | 15_07_2_RNA | NADH dehydrogenase [ubiquinone] flavoprotein 1, mitochondrial | *Neocallimastix californiae* | Unreviewed- protein inferred from homology |
| **A0A1Y1XPP5** | 15_07_1_RNA, 15_07_2_RNA | Alanine--tRNA ligase | *Anaeromyces robustus* | Unreviewed- protein inferred from homology |
| **A0A1Y2E900** | 15_07_1_RNA, 15_07_2_RNA | Dynamin-type G domain-containing protein | *Neocallimastix californiae* | Unreviewed- protein predicted |
| **A0A1Y2ETS6** | 15_07_1_RNA, 15_07_2_RNA | MSF1-domain-containing protein | *Neocallimastix californiae* | Unreviewed- protein predicted |
| **A0A1Y3NVB7** | 15_07_2_RNA | Aconitate hydratase, mitochondrial | *Piromyces sp. (strain E2)* | Unreviewed- protein inferred from homology |
| **P53587** | 15_07_1_RNA, 15_07_2_RNA | Succinate--CoA ligase [ADP-forming] subunit beta, hydrogenosomal | *Neocallimastix frontalis (Rumen fungus)* | Reviewed - Experimental evidence at transcript level |
| **Q7Z941** | ALL | Succinate--CoA ligase [ADP-forming] subunit alpha, mitochondrial | *Neocallimastix patriciarum (Rumen fungus)* | Unreviewed - Experimental evidence at transcript level |

**Table S8: Hydrogenosomal transcripts detected in ZHG gut metatranscriptome.** Hydrogenosomal or malic enzyme encoding protein sequences belonging to Neocallimastigomycetes detected with ShortBRED amongst RNA reads originating from the ZHG microbiome.
